# IL-24 armored CAR19-T cells show enhanced antitumor activity and persistence

**DOI:** 10.1038/s41392-020-00380-8

**Published:** 2021-01-14

**Authors:** Qian Hu, Yuxuan Zhang, Peiyun Wang, Miaojin Zhou, Zhiqing Hu, Cong Liu, Mujun Liu, Lingqian Wu, Xionghao Liu, Desheng Liang

**Affiliations:** 1 Center for Medical Genetics & Hunan Key Laboratory of Medical Genetics, School of Life Sciences, Central South University, Changsha, Hunan China; 2 Hunan Key Laboratory of Basic and Applied Hematology, School of Life Sciences, Central South University, Changsha, Hunan China

**Keywords:** Drug development, Drug development, Translational research, Drug development

**Dear Editor,**

Nowadays, two autologous CAR19-T drugs, Tisagenlecleucel (Kymriah™) and axicabtagene ciloleucel (Yescarta™), have been approved for the treatment of B cell leukemia and lymphoma and achieved unprecedented successes. However, about 10–20% of B-ALL patients receiving CAR19-T drugs didn’t achieve complete remission (CR), while 30~50% of patients achieved CR would relapse mainly within 1 year. Moreover, the high CR rate of CAR19-T therapy for B-ALL can’t be recaptured in other B-NHLs,^1^ such as Burkitt’s lymphoma (BL).^2^ Therefore, there is an urgent need to improve the therapeutic efficacy of CAR19-T cells.^3^

Cytokines play a fundamental role in modulating CAR-T cell functions. Based on our previous antitumor studies with Interleukin-24 (IL-24), we hypothesized that armoring CAR19-T cell with IL-24 might promote its functions. IL-24 is mainly expressed in the immune cells, acting as a potent and near-ubiquitous cancer suppressor in various tumors, such as breast cancer, lung cancer, and lymphoma, etc. Moreover, IL-24 embodied anticancer activity and safety in patients with advanced cancer following direct injection with an adenovirus (Ad.mda-7; INGN-241) into tumors. However, either the effects of IL-24 on CAR19-T cells or the antitumor synergy of IL-24 and CAR19-T cells has not been tested.

In this study, we confirmed that variable concentrations (0.78–200 ng/mL) of recombinant IL-24 protein (rIL-24) significantly inhibited the viability and proliferation of both B-ALL cell line Nalm6 and BL cell line Raji (Supplementary Fig. S1a~d). Moreover, Raji cells treated with rIL-24 showed slower growth in a long-term culture (Supplementary Fig. S1e), which might be due to cell cycle arrest and apoptosis (Supplementary Fig. S1f, g). Then we investigated the effects of IL-24 on human primary T cells, the most commonly used cell resource for CAR-T products. The vitality of T cells isolated from healthy donors by negative sorting was enhanced by approximately 20% at a wide range of rIL-24 treatment (0.78–200 ng/ml) (Supplementary Fig. S2a) without influencing the proliferation (Supplementary Fig. S2b, c). FMC63scFv-28ζ (CAR19), which is frequently used in CAR-T study, was selected as a comparison. Similarly, there was no adverse effects of rIL-24 on either the proliferation (Supplementary Fig. S2d, e) or the ratio and intensity of GFP (reflecting CAR19+) in CAR19-T cells (Supplementary Fig. S2f~h), suggesting the feasibility of armoring IL-24 to CAR19-T cells.

Subsequently, a modified IL-24 cDNA was linked to CAR19 leading to a novel CAR19-IL-24 (Fig. 1a), which was packaged into a lentiviral vector as CAR19. CAR19-IL-24-T cells were prepared under the same process as CAR19-T cells (Supplementary Fig. S3a). Compared to CAR19-T cells, CAR19-IL-24-T cells showed similar expression of CAR19 (Supplementary Fig. S3b) and co-expression pattern of CAR19 and GFP (Fig. 1b), whereas expressed higher level of IL-24 (Supplementary Fig. S3b, c). Besides, the distribution of Th (CD4+) and Tc (CD8+) (Supplementary Fig. S3d, e), GFP+% in the Th and Tc (Supplementary Fig. S3f, g) and the expression of exhaustion-related markers (Supplementary Fig. S3h, i) in CAR19-IL-24-T cells were similar to those in CAR19-T cells. Intriguingly, CAR19-IL-24-T cells possessed more Tn (50% > 30%) and less Teff (30% < 40%) than CAR19-T cells (Supplementary Fig. S3j, k). Collectively, CAR19-IL-24-T cells elevated the expression of IL-24 and tended to be more naive.

**Fig. 1:**
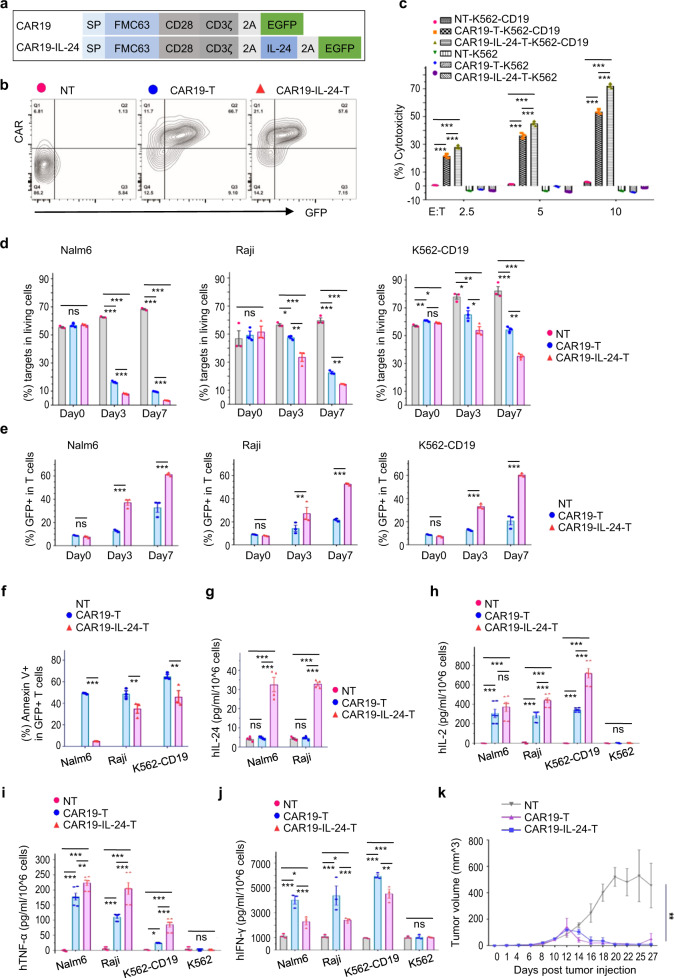
Enhanced antitumor activity and persistence of CAR19-IL-24-T cells. **a** Schematic diagram of FMC63scFv-28ζ (CAR19) and CAR19-IL-24. SP, signal peptide; 2A, 2A “self-cleaving” peptide; EGFP, enhanced green fluorescent protein. **b** Representative flow cytometry data showed co-expression of CAR19 and EGFP in CAR19-T cells and CAR19-IL-24-T cells at days 10 post-transduction. CAR-T cells were confirmed by GFP and labeling with Alexa Fluor 647-AffiniPure F(ab’)2 Fragment Goat Anti-Mouse IgG (H+L) that recognizes scFv portion of the CAR19. **c** The cytotoxicity of T cells to target cells was measured with CytoTox 96® Non-Radioactive Cytotoxicity Assay. T cells were cocultured with K562 cells (CD19−) or K562-CD19 cells (CD19+) for 18 h at the indicated ratios. **d** Quantification and statistical analysis of residue Nalm6 cells, Raji cells, and K562-CD19 cells ratio in living cells subset during coculture. **e** Changes in GFP+ ratio of CAR19-T cells and CAR19-IL-24-T cells after cocultured with tumor Nalm6 cells, Raji cells and K562-CD19 cells at ratio = 1:1, respectively. **f** Quantification and statistical analysis of the apoptosis of GFP+ T cells in CAR19-T group and CAR19-IL-24-T group after challenged with Nalm6 cells, Raji cells, and K562-CD19 cells, respectively. **g**–j T cells were cocultured with tumor cells at ratio 1:1 for 24 h, without exogenous rIL-2. The supernatant was collected for ELISA assay of IL-24 (**g**), IL-2 (**h**), TNF-α (**i**) and IFN-γ (**j**) according to the manufacturer’s suggestions. **k** Analysis of Burkitt lymphoma tumor volumes. NSG mice were inoculated with Raji tumor cells (10^6^ cells/mouse) at day 0, then treated with NT cells, CAR19-T cells and CAR19-IL-24-T cells (normalized to 5% CAR19+, 3 × 10^7^ cells/mouse, *n* = 3/group) at day 8, respectively. All data with error bars were analyzed with GraphPad Prism8 and presented as mean ± SEM. **e**, **f** Unpaired and non-parametric Mann–Whitney test with two-tailed, (**c**, **d**, **g**–**k**) One-way analysis of variance with Bonferroni correction. ns not significant; *p* > 0.05; *p < 0.05, **p < 0.01, ***p < 0.001

To explore whether CAR19-IL-24-T cells enhanced antitumor activity, we tested their short-term cytotoxicity using LDH release experiments. As expected, CAR19-IL-24-T cells showed higher cytotoxicity to Nalm6, Raji, and K562-CD19 cells than CAR19-T cells in a dose-dependent and CD19-specific manner (Supplementary Fig. S4a and Fig. 1c). Furthermore, we evaluated their long-term cytotoxicity using a new Flow-Cytometry-based-Cytotoxicity-Assay (Supplementary Fig. S4b), in which tumor cells were respectively cocultured with NT cells, CAR19-T cells and CAR19-IL-24-T cells for 7 days. CAR19-IL-24-T cells induced apoptosis of Nalm6 cells and Raji cells similar to CAR19-T cells, while triggering a higher apoptosis in K562-CD19 cells (Supplementary Fig. S4c). Since the initial proportion of tumor cells among cocultured mixtures were almost the same, tumor cells cocultured with CAR19-IL-24-T cells showed minimal residue (Supplementary Fig. S4d and Fig. 1d), indicating that CAR19-IL-24-T cells showed stronger competence in eliminating tumor cells. Notably, only CAR19-IL-24-T cells could effectively lower the ratio of K562-CD19 cells (Fig. 1d). Contrasting to Nalm6 cells, Raji cells were relatively insensitive to CAR19-T cells (Fig. 1d), suggesting that BL was more challenging than B-ALL for CAR19-T therapy.

The expansion and survivability of CAR-T cells affect the outcomes of CAR-T therapy. We noticed that CAR19-IL-24-T cells expanded faster than CAR19-T cells (Supplementary Fig. S4e). To determine the enhanced proliferation in responding to tumor, CAR19-IL-24-T cells were cocultured with Nalm6 cells, Raji cells, and K562-CD19 cells, respectively. The GFP+% in the CAR19-IL-24-T group gradually surpassed that in CAR19-T group (Fig. 1e). Generally, the proliferation of CAR19-IL-24-T cells was more robust (Supplementary Fig. S4f). In short, CAR19-IL-24-T cells improved proliferation irrespective of tumor stimulus, implying a better persistence in vivo. We further investigated the apoptotic fate of CAR19-IL-24-T cells. Both NT cells and CAR19-T cells were high apoptotic after cocultured with tumor cells, while the apoptotic rate of CAR19-IL-24-T cells was significantly lower (Fig. 1f and Supplementary S4g, h), indicating that CAR19-IL-24-T cells were more anti-apoptotic, which would contribute to their survival and antitumor potential under the pressure of tumor cells.

Clinical data showed that IL-24 was downregulated in tumor tissues and low IL-24 expression was identified as a predictor of poor prognosis in BL patients.^4^ In our study, we observed that CAR19-IL-24-T cells secreted IL-24 eight times higher than CAR19-T cells (Fig. 1g). The safety concern that CAR19-IL-24-T cells would elevate the level of IL-24 in the entire body and then evoke unexpected side effects could be addressed by using adjustable expression strategies. Knowing that IL-24 is a multidimensional anti-cancer therapeutic, such as transforming the TME by promoting T cells infiltration, IL-24 secreted by CAR19-IL-24-T cells would affect both tumor cells and T cells, which in turn enhance the antitumor ability of CAR19-IL-24-T cells. Therefore, targeted delivery of IL-24 by CAR19-IL-24-T cells would be more precise and effective compared to systemic or local administration of rIL-24. Moreover, CAR19-IL-24-T cells produced higher levels of both IL-2 and TNF-α and lower level of IFN-γ compared to CAR19-T cells (Fig. 1h~j). A lower level of IFN-γ might be safer according to previous reports.^5^

We preliminarily evaluated the short-term efficacy and safety of CAR19-IL-24-T cells in vivo (Supplementary Fig. S5a). CAR19-IL-24-T cells reduced BL tumor volumes in M-NSG mice as effectively as CAR19-T cells (Fig. 1k). Besides, no obviously unfavorable effects on the body weight were observed after a large dose of CAR19-IL-24-T cells treatment (Supplementary Fig. S5b). We analyzed the embedded T cells in the blood of xenograft mice (Supplementary Fig. S5c), finding a higher T cell population and more GFP+T cells in the CAR19-IL-24-T treatment group compared to CAR19-T treatment group although there was no statistical significance (Supplementary Fig. S5d~f). We assumed that IL-24 incurred profound impacts on CAR19-IL-24-T cells by providing with a unique expression profile of key regulators that involved in autophagy and RNA epigenetic modification (Supplementary Fig. S5g). Next, we will employ the xenograft model to explore the antitumor mechanisms of CAR19-IL-24-T cells and evaluate the potential side effects. It is also needed to understand the limitations and meet the challenges for extending this approach to other tumors.

In summary, we provided a successful proof of concept for IL-24 armored CAR19-T cells to enhance antitumor efficiency, while improving proliferation and persistence with anti-apoptotic properties. Moreover, CAR19-IL-24-T cells secreted excess IL-24 as a powerful weapon to antitumor synergistically. In brief, CAR19-IL-24-T cells exhibit potential in clinical application, suggesting a new path forward to the improvement of CAR-T therapy.

## Supplementary information

Supplementary Materials for IL-24 armored CAR19-T cells show enhanced antitumor activity and persistence
