# Supplementary material for: IL-24 armored CAR19-T cells show enhanced antitumor activity and persistence
Source: Signal Transduct Target Ther. 2021 Jan 14;6:14. doi: 10.1038/s41392-020-00380-8 (PMC7806903; doi:10.1038/s41392-020-00380-8)
Supplement: Supplementary file 1 — Supplementary Materials for IL-24 armored CAR19-T cells show enhanced antitumor activity and persistence [file 41392_2020_380_MOESM1_ESM.docx]

Supplementary Materials for

IL-24 armored CAR19-T cells show enhanced antitumor activity and persistence

Qian Hu^1^, Yuxuan Zhang^1^, Peiyun Wang^1^, Miaojin Zhou^1^, Zhiqing Hu^1^, Cong Liu^1^, Mujun Liu^1^, Lingqian Wu^1^, Xionghao Liu^1,2^***** and Desheng Liang^1,2^*****

^1^Center for Medical Genetics & Hunan Key Laboratory of Medical Genetics, School of Life Sciences, Central South University, Changsha, Hunan, China

^2^Hunan Key Laboratory of Basic and Applied Hematology, School of Life Sciences, Central South University, Changsha, Hunan, China

Correspondence to: Xionghao Liu ([liuxionghao@sklmg.edu.cn](mailto:liuxionghao@sklmg.edu.cn)) and

Desheng Liang (liangdesheng@sklmg.edu.cn)

**This PDF file includes:**

Materials and Methods

Figures. S1 to S5

Materials and Methods

**1. Cell lines**

HEK 293T cell lines were cultured in DMEM (Gibco, Carlsbad, CA, USA ) supplemented with 10% FBS (Gibco) (shortly, complete DMEM medium) for the production of lentivectors. Raji (Burkitt's lymphoma cell line, which endogenously express CD19), Nalm-6 (B-ALL cell line, which endogenously express CD19), K562 (AML cell line, CD19-) and K562-CD19 (overexpressing hCD19, gifted from Dr. Jie Liu) tumor cells were cultured in RPMI 1640 (Gibco) supplemented with 10% FBS (Gibco), 1% nonessential amino acids (Invitrogen, Carlsbad, CA, USA), 2 mmol/L L-glutamine (Invitrogen) and 1% sodium pyruvate (Invitrogen) (shortly, complete RPMI1640 medium). All cell lines were regularly validated to be mycoplasma-free by PCR analysis. Cell culture was performed at 37 °C in a humidified atmosphere with 5% CO_2_.

**2. Design and construction of CAR plasmids**

To make CAR19-IL-24 expressing lentiviral vectors, we first synthesized a CAR19 fragment (a CD19 specific scFv derived from FMC63 clone was fused to the intracellular domains from human CD28 and CD3ζ) flanked by BamH1/Age1 sites. Then, CAR19 fragment was inserted into a BamH1/Age1-digested 2^nd^ lentiviral vector pHQ-hPGK-eGFP, resulting in the new plasmid named as pHQ-hPGK-CAR19-eGFP. After that, an optimized hIL-24 cDNA fragment modified from our prior work was cloned into pHQ-hPGK-CAR19-eGFP to construct pHQ-hPGK-CAR19-IL-24-eGFP. All lentiviral vectors were verified by Sanger sequencing and transformed into Stbl3 Chemically Competent *E.coli* for the purification of advanced transfection-grade plasmid using EndoFree Plasmid Maxi Kits (QIAGEN,12362). The aliquots of transfection-grade plasmid were stored at -80 °C for the preparation of lentiviruses.

**3. Production of CAR-encoding recombinant lentiviruses**

Before the production of lentiviral particles, all plasmids were sequenced in both directions to confirm CAR or other element sequences. Briefly, ten million HEK 293T cells in logarithmic growth phase were seeded into T75 flasks and cultured overnight, then co-transfected with the pMD2.G (Addgene 12259), pSPAX2 (Addgene 12260) Lentivector Packaging mix and 10 μg of lentiviral vectors per T75 flask using the FuGene HD Transfection Kit (Promega, E2311). The medium was replaced with fresh complete DMEM medium at post-transfection 6 h. The culture supernatants containing recombinant lentiviruses were harvested at 24 h, 48 h and 72 h after transfection, and cleared of cell debris by centrifugation at 2500 g for 20 min and 0.45-μm filtration (Merck & Co Inc., NJ, USA). The virus particles were collected by high-speed centrifugation at 112,000 g for 180 min using Beckman Ultracentrifuge and suspended in AIM V medium (Gibco, 12055083), aliquoted and frozen at -80 °C. The lentiviral titers were measured on HEK 293T cells based on the ratio of GFP-positive cells by flow cytometry, and were > 0.5x10^8 TU/ml. Lentiviruses were generated and used under approved biosafety level-2 regulations.

**4. Generation of CAR-T cells**

This study was conducted according to the principles of the Declaration of Helsinki and with the approval of the Scientific Ethics Committee of the CSU. Written informed consent was obtained from all subjects after oral explanation of this study. Human peripheral blood mononuclear cells (PBMCs) from healthy individuals were prepared by Histopaque-1077 (Sigma,10771) density gradient centrifugation and cultured in complete RPMI1640 medium supplemented with 300 IU/ml rIL-2 (Peprotech, Rocky Hill, NJ, USA; 200-02) at tissue-treated 10cm dishes about 6 h to remove plastic-adherent monocytes. T cells were negatively selected from monocyte-depleted PBMCs using the Pan T cell isolation kit (Miltenyi Biotec, Germany; 130-096-535) as the manufacturer's instructions. The purity of isolated pan T cells was assayed using APC conjugated anti-human CD3 (BioLegend). Freshly isolated pan T cells were activated by anti-CD3/CD28 antibody-coated beads (at a Cell:Bead ratio=2:1) (Invitrogen, 11161D) and cultured in AIM V medium (Gibco, 12055083) supplemented with 5% FBS (Gibco) (shortly, complete AIM V medium) and 100 IU/ml rIL-2 (Peprotech, 200-02) for 24 h. On day 0, activated T cells were transduced by the appropriate amount of concentrated lentiviral supernatants (at a multiplicity of infection [MOI] of 20) supplemented with 0.8 μg/ml of polybrene. Six hours later, 1 ml fresh complete AIM V media containing 300 IU rIL-2 (Peprotech, 200-02) was added to each well. Donor-matched activated, but non-transduced T (NT) cells were expanded to serve as a negative control in subsequent assays.

As the T cells proliferated over the next two weeks, the cells were counted with hemocytometer every 2-3 days and fresh complete AIM V medium with 300 U/ml rIL-2 (Peprotech, 200-02) was added to the cultures to maintain the cell density at 0.5~1 × 10^6 cells/ml. Once T cells appeared to rest down, as determined by both decreased growth kinetics and cell size, they were either used for functional assays or cryopreserved. The images of cell morphology were acquired with an optical microscope fitted with a camera (Leica, Germany). Usually at days 7 to 14 of culture, CAR19 expression was measured using Alexa Fluor 647 conjugated Goat Anti-Mouse IgG F(ab’)2 (Jackson ImmunoResearch, USA, 115-606-003) or reflected by GFP expression indirectly, and NT cells as negative control. Then, T cells were de-beaded and rested overnight for functional assays. T cells also were cryopreserved after de-beaded for a backup or long-term preservation. If not otherwise indicated, all T cell functional assays were performed in media without cytokines.

**5. Western Blot**

Cells (~5×10^6) were washed twice with ice-cold DPBS resuspended in 150 μL radioimmunoprecipitation assay (RIPA) buffer supplemented with Protease Inhibitor Cocktail (Sigma, P8340) and 1 mM PMSF, and incubated on ice for 30 min. The cell lysates were cleared by centrifugation at 4 °C, 16,000 g for 30 min and determined the concentrations of proteins using a BCA protein assay kit (Pierce Biotechnology, USA) and mixed with 5X sample loading buffer and boiled at 99 °C for 10 min. Boiled samples were separated by electrophoresis in a 5%-12% SDS-PAGE gel and transferred to an Immobilon P membrane (Merck & Co Inc., NJ, USA). The membrane was blocked with the blocking buffer (5% non-fat dried milk and 0.1% Tween-20 in Tris-buffered saline [TBS]) at RT for 60 min. The blocked membrane was incubated at 4°C overnight with mouse anti-GAPDH antibody (Sigma, G9295, 1:5,000 dilution) or mouse anti-human CD3ζ antibody (BD, USA; 551033, 1:5000 dilution) or goat anti-human IL-24 antibody (R&D, AF1965, 1:2000 dilution) diluted in a blocking buffer. The membrane was then incubated for 1 h with anti-goat immunoglobulin G (IgG)-horseradish peroxidase (HRP) (Jackson Lab, 805-035-180, 1:10,000 dilution) or anti-mouse IgG-HRP (Sigma, A2554, 1:10,000 dilution) diluted in a blocking buffer. The membrane was washed three times with 0.1% Tween-20/TBS after per antibody incubation steps. Immunoreactivity was visualized with a Pierce™ ECL Plus (Thermo Fisher) as a substrate and detected with ChemiDoc XRS+ (Bio-Rad) as per the manufacturer's instructions. Results were confirmed by at least three independent experiments.

**6. Quantitative real-time PCR**

RNA was extracted with TRIzol reagent (Invitrogen, 15596018) and RNA quality and quantity were determined using a NanoDrop 2000 (Thermo Fisher). RNA was reverse transcribed into cDNA using HiScript® II Q Select RT SuperMix (Vazyme, China; R233-01) and processed for quantitative real-time PCR using a CFX96 real-time PCR detection system (Bio-Rad) using the ChamQ Universal SYBR qPCR Master Mix (Vazyme, Q711). The results were normalized to the level of 18s, and the relative expression levels were calculated using the 2–ΔΔCT method. The following primers were used for the real-time PCR experiments:

| Ribosomal 18S: F AACCCGTTGAACCCCATT |
| --- |
| Ribosomal 18S: R CCATCCAATCGGTAGTAGCG |
| IL-24: F CAGGCGGTTTCTGCTATTC |
| IL-24: R GAATTTCTGCATCCAGGTCA |
| T-bet: F GAAACCCAGTTCATTGCCGT |
| T-bet: R CCCCAAGGAATTGACAGTTG |
| EOMES: F ACT GGTTCCCACTGGATGAG |
| EOMES: R CCACGCCATCCTCTGTAACT |
| GATA3: F CACAACCACACTCTGGAGGA |
| GATA3: R GGTTTCT GGTCTGGATGCCT |

**7. Flow Cytometry**

T cells were stained for cell-surface markers to determination of phenotypes with the following antibodies: CD3 (APC; BioLegend), CD4 (PE; BioLegend), CD8 (PerCP/Cy5.5; BioLegend), CD19 (FITC and PE; BD), TIM-3 (PE; BioLegend), LAG-3 (PcrCP/Cy5.5; Biolegend), PD-1 (APC; BioLegend), CD45RA (BV510; BD), CCR7 (BB700; BD). Exhaustion of the CAR-T cells was analyzed with TIM-3, LAG-3 and PD-1. CD45RA and CCR7 can be used to evaluate the differentiated state of T cells by using the following expression patterns: Tn (naïve T cells), CD3+CD45RA+CCR7+; Tcm (central memory T cells), CD3+CD45RA−CCR7+; Tem (effector memory T cells), CD3+CD45RA−CCR7−; Teff (effector T cells), CD3+CD45RA+CCR7−. To measure CAR expression or transduction efficacy, 0.5 million cells were washed once and suspended in 100 μl of buffer (PBS containing 0.5% BSA) and incubated on ice with polyclonal goat anti-mouse F(ab)’2 antibody (Alexa Fluor 647, Jackson Immunoresearch, 115-606-003) for 30 min. CD3+F(ab)2+ or CD3+GFP+ cells were defined as CAR+ cells, NT cells served as negative control. For further characterization, cells were washed and re-suspended in cell staining buffer, incubated for 30 min with the above antibodies on ice, washed and measured using FACS Calibur (BD), FACS Canto II (BD) or DxP Athena™ (Cytek). Data analysis was performed using FlowJo (Treestar, Ashland, OR).

Cell Proliferation was measured by cell tracking dye-dilution in T cells and tumor cells using CellTrace™ CFSE Cell Proliferation Kit (Invitrogen, C34554) or Tag-it Violet™ Proliferation and Cell Tracking Dye (Biolegend, 425101). To analysis of cell cycle, 10^6 cells were washed once and fixed in cold 70% ethanol at 4 °C overnight. Then, fixed cells were stained with Propidium iodide (PI) (Biolegend, 421301) according to the manufacturer’s instructions. To detect apoptosis, 10^6 cells were washed once and stained with Annexin V Apoptosis/Detection kit (BD) following the guidance of the manufacturer’s protocol.

**8.** **Co-culture cytotoxicity assay**

The number of CAR+ cells was obtained using the cell counts from the hemocytometer and the CAR+ or GFP+ population percentages obtained from flow cytometry. In cases of unequal percentages of CAR+ cells in the cultures, same batch donor matched NT cells were added to ensure that both the number of CAR+ T cells and the total number of T cells remained consistent across the CAR19-T and CAR19-IL-24-T groups. Then they were used in the following experiments.

**Lactate dehydrogenase release-based cytotoxicity assay**

To determine the cytotoxicity of the CAR-T cells to K562-CD19 cells and K562 cells, target cells (1 × 10^4) were seeded in each well of a 96-well V-bottom plate (Corning, USA). The CAR-T cells were added at different effector-target ratios (2.5, 5 and 10) and then co-cultured in complete RPMI1640 medium without cytokines. After 18 h incubation, the cell-free supernatant was collected by centrifugation. Lactate dehydrogenase (LDH) release in the supernatant was assayed using CytoTox 96® Non-Radioactive Cytotoxicity Assay (Promega, G1780). The cytotoxicity was calculated according to the manufacturer’s instructions.

**Flow cytometry-based cytotoxicity assay (FCbCAssay)**

To test interaction and toxicity between T cells and tumor cells during coculturing, we designed a Flow cytometry-based cytotoxicity assay (FCbCAssay). Briefly, at day 0, T cells labeled with Tag-it Violet™ dye (Biolegend, 425101) and tumor target cells labeled with CellTrace™ Far Red (Invitrogen, C34572) were mixed at the ratio 1:1 and cocultured in complete RPMI1640 medium without rIL-2. T cells were removed anti-CD3/28 beads and then cultured in RPMI1640 medium without rIL-2 for resting 24h before labeled. Aliquots of well-mixed cultures were seed in a 24-well plate, and tested using Flow cytometry on days 0, 3 and 7. Specifically, Cells were harvested and washed once with DPBS and stained using Zombie NIR Fixable Viability Kit (Biolegend, 423105). Percentage of viable target tumor cells in total living cells subset (gated Zombie NIR-) was analyzed as an indicator of direct cytotoxicity of T cells against tumor cells. In addition, Tag-it Violet-dilution in T cells (represents the degree of T cell proliferation), CAR+ ratio (also mirrored by GFP) and the apoptosis of T cells or tumor cells (stained by Annexin V) were also monitored.

**9. ELISA assays cytokine production**

For ELISA assays to test cytokine production by CAR-T cells, CD19+ (Nalm6, Raji or K562-CD19) or CD19- (K562) target cells were cocultured with NT cells, CAR19-T cells and CAR19-IL-24-T cells at a ratio of 1:1 (1×10^5 each) in a total of 200 µl medium and seeded in duplicate wells of a 96 well round-bottom plate (Corning) in complete RPMI1640 medium without rIL-2. The plates were incubated at 37 °C for 24 hours. Following the incubation, the culture supernatants including cells were harvested and centrifuged to obtain cell-free supernatants. ELISAs for IL-2, TNF-α, IFN-γ and IL-24 were performed by using standard methods (R&D Systems, Inc. Minneapolis, USA). Measurements were performed in triplicates.

**10. Xenograft models of human Burkitt lymphoma (BL)**

Mouse experiments were approved by the CSU animal care and use committee. Xenograft tumors were established by subcutaneous injection of 1 x 10^6^ Raji cells. Raji tumors were allowed to grow in M-NSG mice (NOD-*Prkdc*^scid^*Il2rg*^em1^/Smoc, Shanghai Model Organisms Center, Inc., 6~8-week-old, about 24 g) for 8 days. Mice were then treated with tail-vein injection of 3 x 10^7^ CAR-T cells ( Both CAR19-T cells and CAR19-IL-24-T cells were adjusted with NT cells for the percentage of CAR+ cells in the final products, normalized to 5% CAR19 expression, n = 3/group) at day 8. Tumor dimensions were measured with calipers, and tumor volumes calculated using the formula V= 1⁄2 x L x W x W, where L is length (longest dimension) and W is width (shortest dimension). Tumor volume, body weight and other CRS-like symptoms (malaise, piloerection) were monitored. For monitoring of CAR-T cell expansion in mice, peripheral blood samples were collected via tail clip for quantification of [embedded](javascript:;) T cells by flow cytometry.

**11. Graphs and Statistical Analysis**

All statistical analysis in this study was performed using Prism8 (GraphPad Software). As stated in the figure legends, ANOVA with Bonferroni correction and unpaired and non-parametric Mann-Whitney test with two-tailed were used for comparison of 3 or more groups, or 2 groups respectively. Data are presented as mean ± SEM and p < 0.05 was considered statistically significant, and p values are denoted with asterisks as follows: ns, not significant, p > 0.05; * p < 0.05, ** p < 0.01, *** p < 0.001.

Figure. S1.


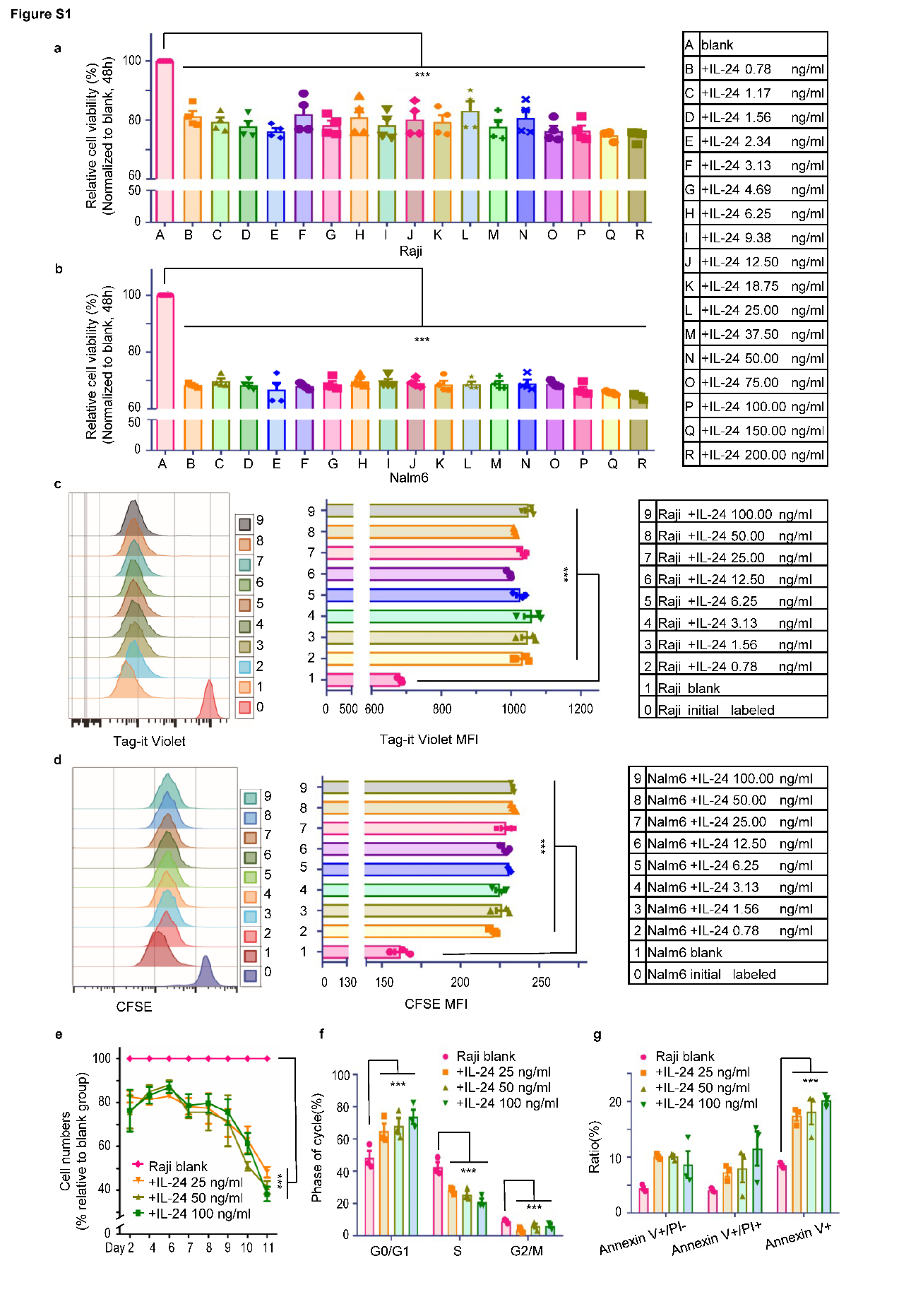


**Fig S1. rIL-24 inhibits Burkitt lymphoma Raji cells and B-ALL Nalm6 cells in vitro.**

(a,b) Tumor cells were treated with variable concentrations (0.78~200 ng/ml) of rIL-24 for 48 h and then the cell viability was measured by CCK8 assay. The viabilities of both Raji cells (a) and Nalm6 cells (b) after rIL-24 treated were significantly less than the control group.

(c) Cell Proliferation of Raji cells was measured by Tag-it Violet-dilution. Raji cells were labeled with Tag-it Violet™ dye and then treated with varying concentrations (0.78 doubled up to 100 ng/mL) of rIL-24 for 5 days. Fresh medium with corresponding rIL-24 concentration were supplemented every other day.

(d) Cell Proliferation of Nalm6 cells was measured by CFSE-dilution. Nalm6 cells were labeled with CellTrace™ CFSE dye and then treated with varying concentrations (0.78 doubled up to 100 ng/mL) of rIL-24 for 5 days. Fresh medium with corresponding rIL-24 concentration were supplemented every other day.

(e~g) Raji cells were cultured in media without or supplemented with 25, 50 and 100 ng/mL rIL-24, respectively. Analysis of Raji’s cell number (e), cell cycle (f) and apoptosis (g) at days 10 of culture.

All data with error bars were analyzed with GraphPad Prism8 and presented as mean ± SEM. One-way analysis of variance, Bonferroni's Multiple Comparison Test, * p < 0.05; ** p < 0.01; *** p < 0.001.

Figure. S2.


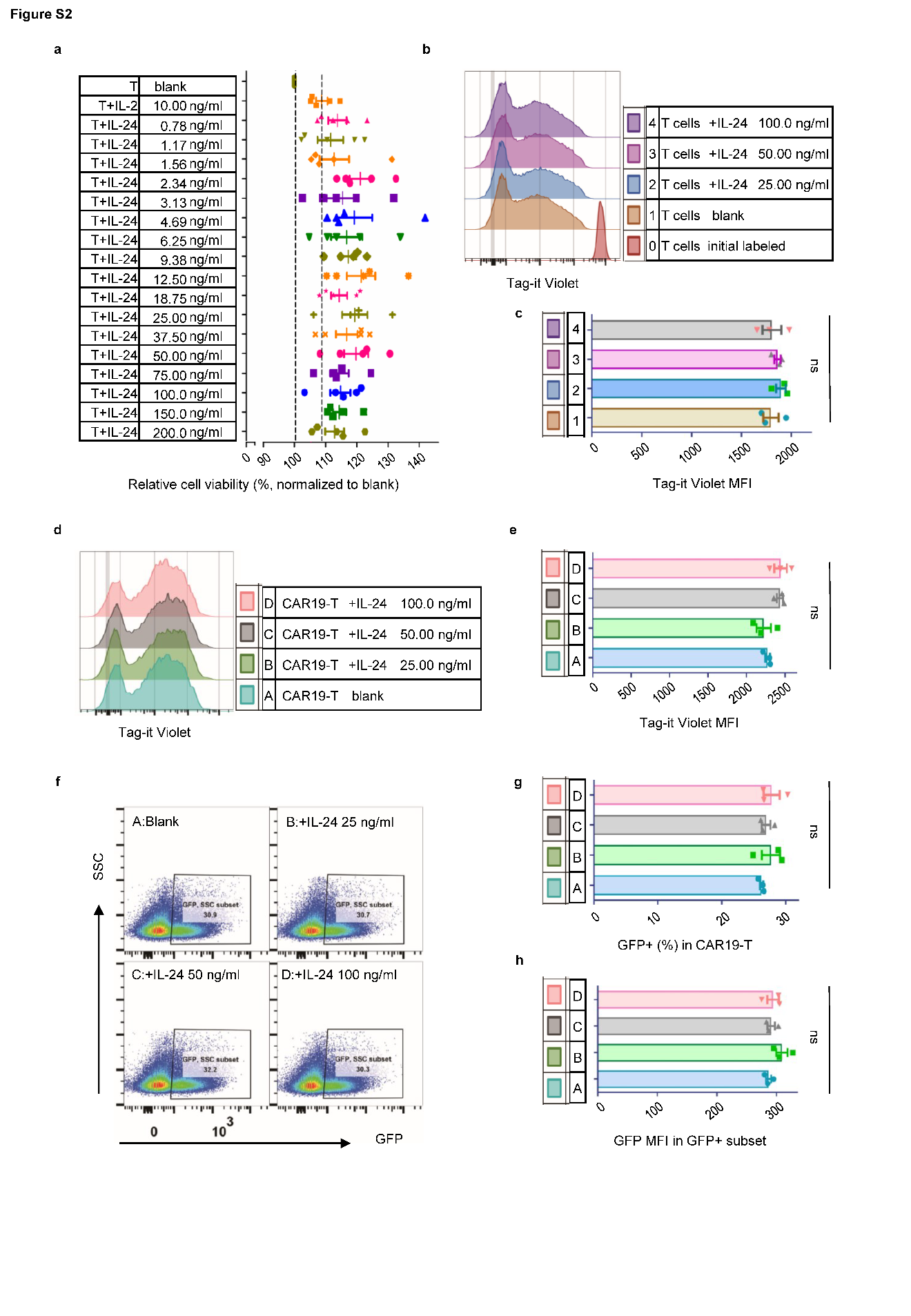


**Fig S2. Varying concentrations (0.78~200 ng/ml) of rIL-24 did not inhibit both T cells and CAR19-T cells.**

(a) Human primary pan T cells freshly isolated from healthy donors by negative sorting were treated with varying concentrations (ranging from 0.78~400 ng/mL) rIL-24 in absence of rIL-2 for 2 days. The viabilities of T cells were measured by CCK8 assay.

(b, c) Cell Proliferation of pan T cells was measured by Tag-it Violet-dilution. Freshly isolated human primary pan T cells were expanded and de-beads, cultured in RPMI1640 medium without rIL-2 for resting 24 hours. Then they were labeled with Tag-it Violet™ dye and treated with rIL-24 (25, 50 or 100 ng/ml) for 9 days. Representative flow cytometry data (b) and statistical results (c) of MFI of Tag-it violet in T cells.

(d~h) CAR19-T cells were labeled with Tag-it Violet™ dye and treated with rIL-24 (25, 50 and 100 ng/ml) for 9 days.

(d) Representative flow cytometry data showed Tag-it violet-dilution in CAR19-T cells and (e) Statistical results of MFI of Tag-it violet in CAR19-T cells.

(f) Representative flow cytometry data showed GFP ratio in CAR19-T cells.

(g) Changes in frequency of GFP+% and MFI of GFP (h).

All data with error bars were analyzed with GraphPad Prism8 and presented as mean ± SEM. One-way analysis of variance, Bonferroni's Multiple Comparison Test, ns, no statistically significant; p > 0.05.

Figure. S3.


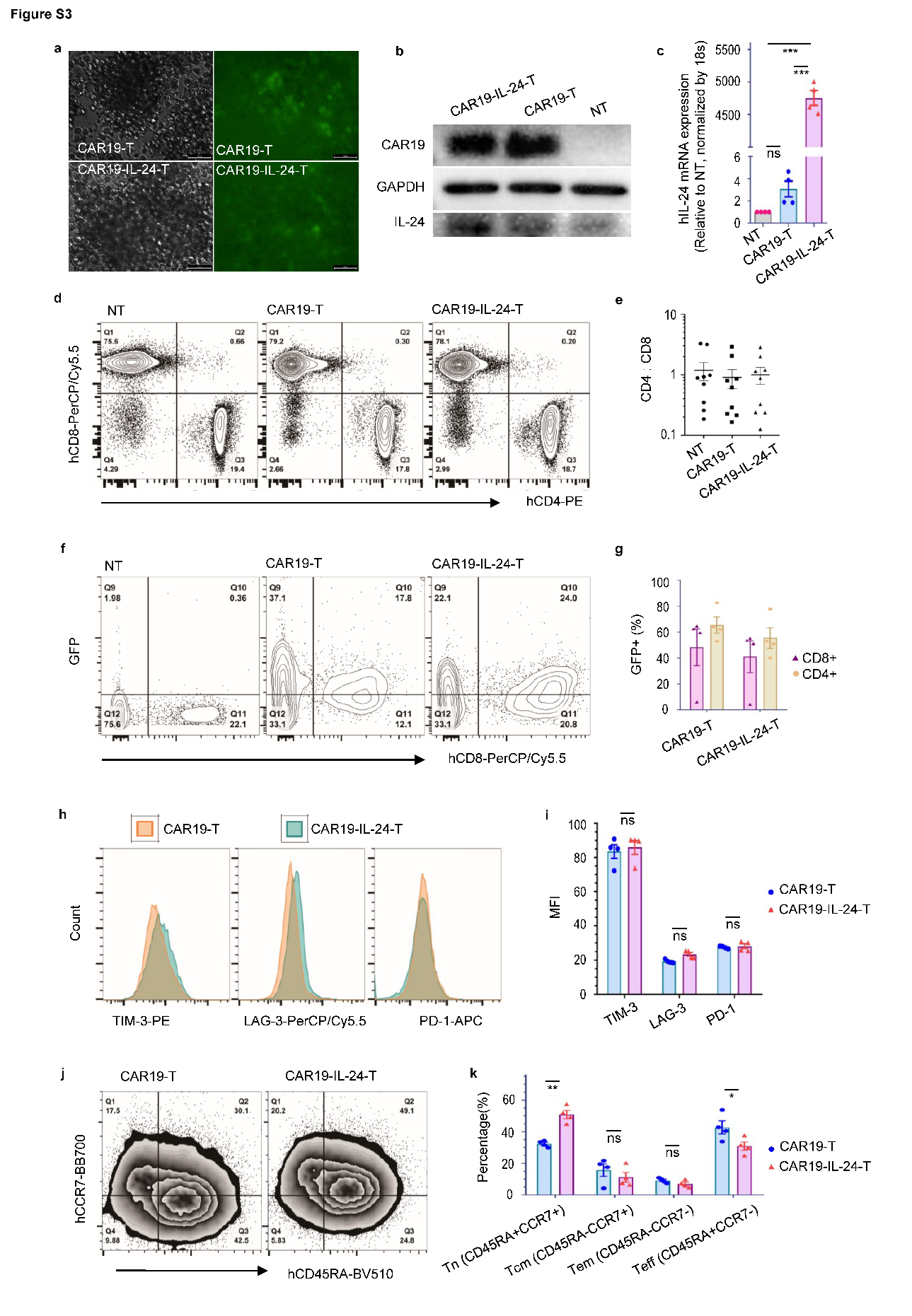


**Fig S3. Characterization of CAR19-IL-24-T cells.**

(a) Cell morphologies of CAR19-T cells (up) and CAR19-IL-24-T cells (down) were captured with inverted fluorescence microscope at days 7 post-transduction.

(b) Representative Western blot showed the protein levels of CAR19 and IL-24 in CAR19-T cells and CAR19-IL-24-T cells. CAR19 was probed by anti-hCD3ζ antibody (1:5000 dilution); IL-24 was probed by anti-hIL-24 antibody (1:2000 dilution). Data represent at least in 3 independent experiments.

(c) Analysis of the mRNA level by qPCR.

(d, e) The Tc (CD8+) and Th (CD4+) distribution in T cells.

(d) Representative flow cytometry plot showing CD4 and CD8 expression gated in CD3+ subset in NT cells, CAR19-T cells and CAR19-IL-24-T cells and (e) frequency of CD4+ or CD8+ at day 10 post-transduction.

(f, g) GFP signal (reflecting CAR19 expression) in CD4+ or CD8+ T cells.

(f) Representative flow cytometry showing GFP and CD8 expression gated in CD3+ subset.

(g) Frequency of GFP+ in CD4+ and CD8+ subset at day 10 post-transduction.

(h, i) Flow cytometry analysis and quantification of the T cell exhaustion-associated surface markers TIM-3, LAG-3 and PD-1.

(h) Histogram showing TIM-3, LAG-3 and PD-1 expression in CAR19-T cells and CAR19-IL-24-T cells and (i) Quantification and statistical analysis of the data in (h).

(j, k) Surface expression of CD45RA and CCR7 to distinguish differentiation status: Tn (naïve, CD3+CD45RA+CCR7+), Tcm (central memory, CD3+CD45RA−CCR7+), Tem (effector memory, CD3+CD45RA−CCR7−) and Teff (effector, CD3+CD45RA+CCR7−).

(j) Flow cytometry showing representative expression of CD45RA and CCR7 in CAR19-T cells and CAR19-IL-24-T cells at day 10 post-transduction and (k) relative frequency of four differentiation status.

All data with error bars were analyzed with GraphPad Prism8 and presented as mean ± SEM.

(c, e) One-way analysis of variance with Bonferroni correction. (g, i and k) unpaired and non-parametric Mann-Whitney test with two-tailed, ns, not significant; p > 0.05; * p < 0.05, ** p < 0.01, *** p < 0.001.

Figure. S4.


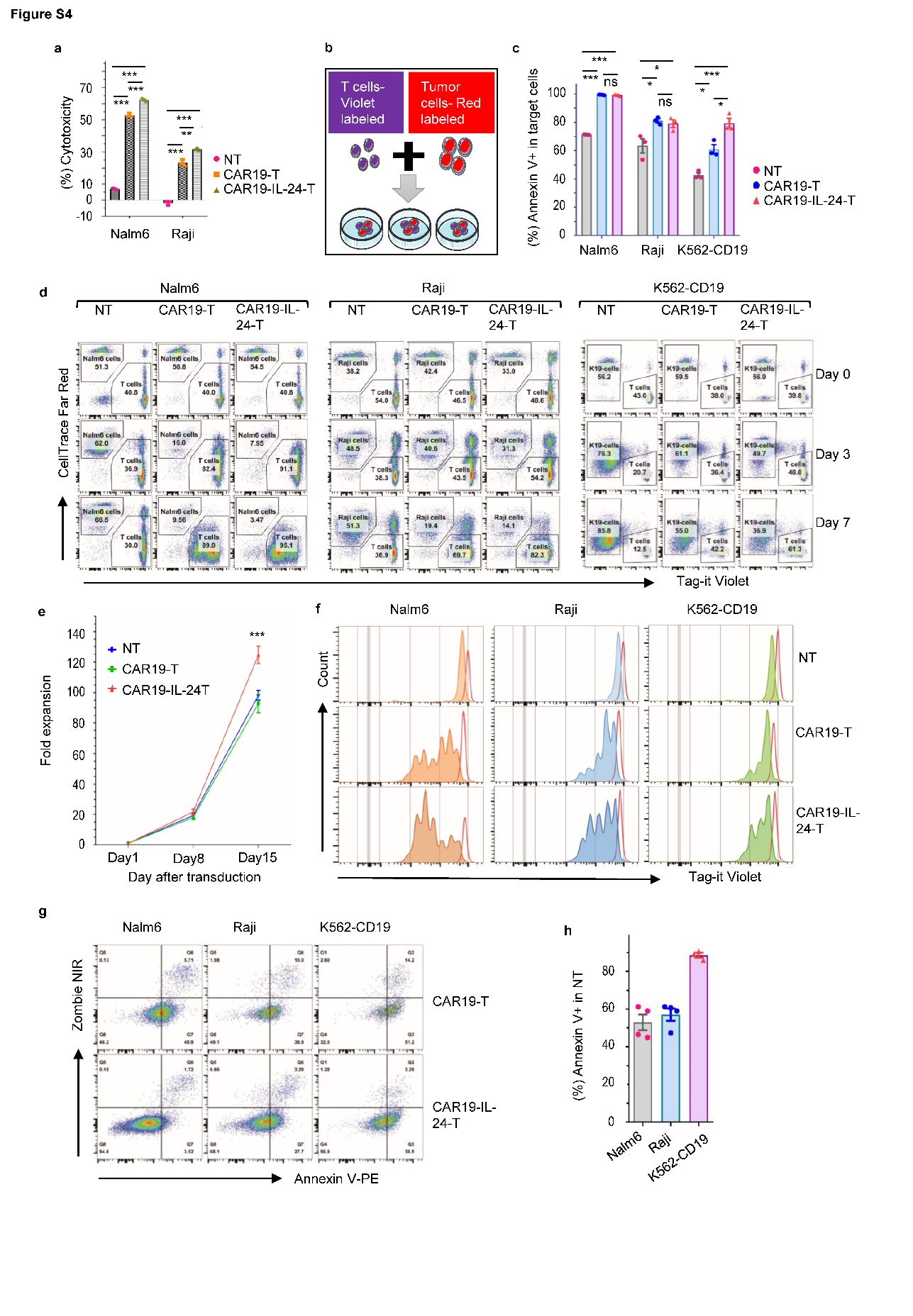


**Fig S4.** **CAR19-IL-24-T cells showed enhanced antitumor activity and improved proliferation with anti-apoptotic properties.**

(a) The cytotoxicity of T cells to target cells was measured with CytoTox 96® Non-Radioactive Cytotoxicity Assay. T cells were cocultured with Nalm6 cells or Raji cells for 18h at the ratio of 2.5 : 1 .

(b) Schematic diagram of flow-cytometry-based-cytotoxicity-assay (FCbCAssay) for direct evaluating cytotoxicity of effectors against targets. T cells labeled with Tag-it Violet™ dye and tumor cells labeled with CellTrace™ Far Red were mixed at the ratio 1:1 and coculture in complete RPMI1640 medium without rIL-2. T cells were removed anti-CD3/28 beads and then cultured without rIL-2 for resting 24h before labeled.

(c) Quantification and statistical analysis of the apoptosis of Nalm6 cells, Raji cells and K562-CD19 cells induced by T cells, by Annexin V stain at day 7 of coculture.

(d) Representative flow cytometry data showed residue tumor cells and T cells gated in living cells subset (Zombie NIR-) during coculture.

(e) Expansion kinetics of NT cells, CAR19-T cells and CAR19-IL-24-T cells post-transduction.

(f) Representative flow cytometry data showed Tag-it Violet-dilution in NT cells, CAR19-T cells and CAR19-IL-24-T cells, respectively. T cells were labeled with Tag-it Violet and then respectively stimulated by Nalm6 cells, Raji cells and K562-CD19 cells at 1:1 ratio for 7 days in absence of rIL-2.

(g) Representative flow cytometry data showed the apoptosis in NT cells and CAR19+ T cells after challenged with Nalm6 cells, Raji cells and K562-CD19 cells, by Annexin V stain at day7 of coculture.

(h) Quantification of the apoptosis in NT cells after challenged with Nalm6 cells, Raji cells and K562-CD19 cells, respectively.

All data with error bars were analyzed with GraphPad Prism8 and presented as mean ± SEM. One-way analysis of variance with Bonferroni correction. ns, not significant; p > 0.05; * p < 0.05, ** p < 0.01, *** p < 0.001.

Figure. S5.


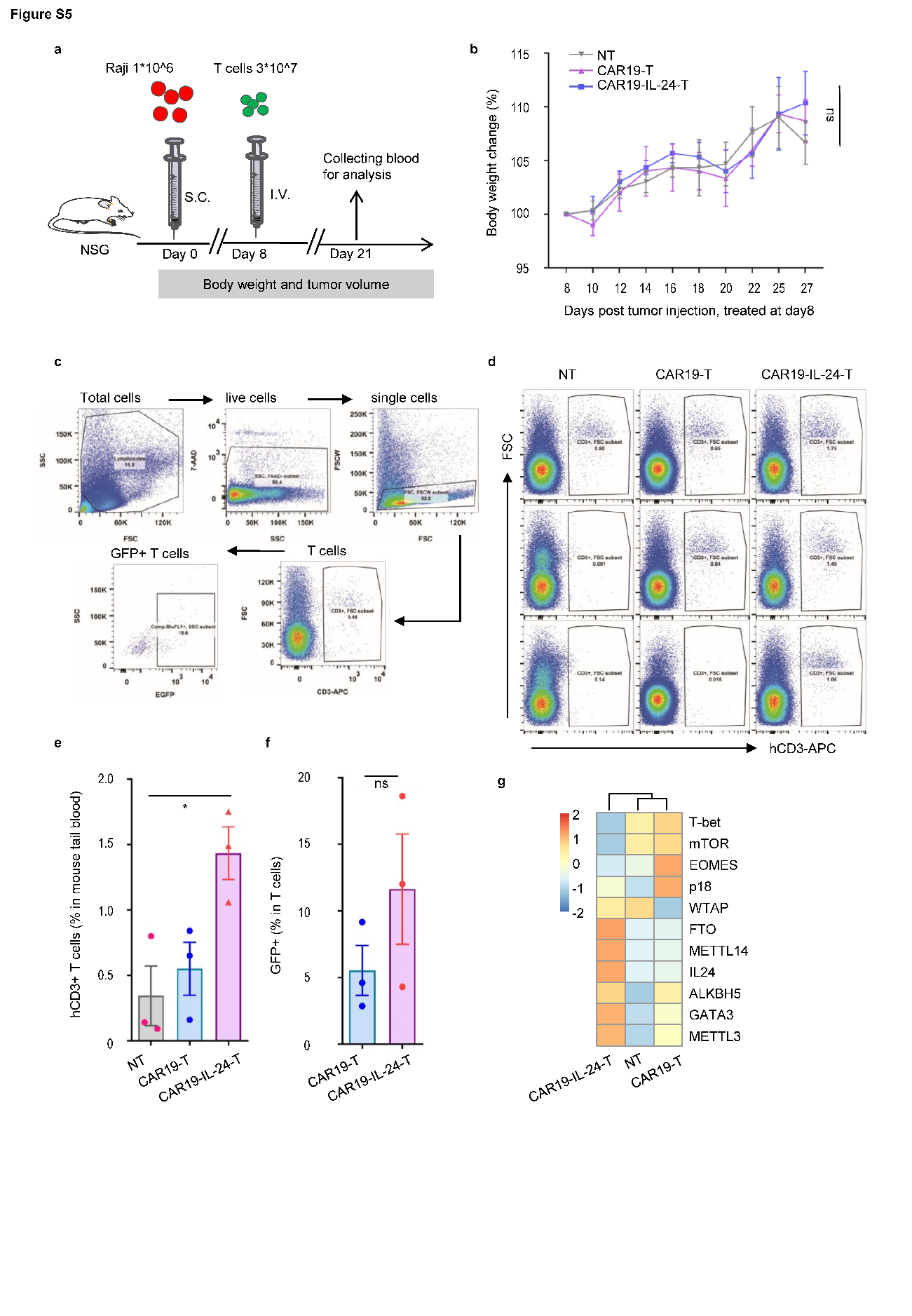


**Fig S5. CAR19-IL-24-T cells showed safety and efficiency in vivo.**

(a) Schema of the xenograft model infused with CAR-Ts on day 8 after tumor inoculation. NSG mice were inoculated with Raji tumor cells (10^6 cells/mouse) at day0, then treated with NT cells, CAR19-T cells and CAR19-IL-24-T cells (normalized to 5% CAR19+, 3*10^7 cells/mouse, n = 3/group), respectively. Tumor volume and body weight was recorded. Blood from tail was collected for analyzing T cells.

(b) Summary of the body weight (day 8~29 after Tumor cell infusion, normalized to body weight of day8 ).

(c) Schema of the analyzing CAR-T cell in peripheral blood samples of mice (day 21 after Tumor cell infusion).

(d) Flow cytometry showing human CD3+ T cells in peripheral blood samples of mice.

(e) Quantification and statistical analysis of the embedded human T cell in (d).

(f) Quantification and statistical analysis of GFP+% in embedded human T cells.

(g) Heat map of T-bet, EOMES, GATA3 and other key regulators such as autophagy (mTOR, p18) and RNA epigenetic modification (METTL3, METTL14, ALKBH5, FTO and WTAP). QPCR data from at least 3 independent experiments.

All data with error bars were analyzed with GraphPad Prism8 and presented as mean ± SEM.

(b, e) One-way analysis of variance with Bonferroni correction, (f) unpaired and non-parametric Mann-Whitney test with two-tailed, ns, not significant; p > 0.05; * p < 0.05, ** p < 0.01, *** p < 0.001.
